# Clinical Evaluation of Transarterial Infusion Chemotherapy for Advanced Esophageal Cancer

**DOI:** 10.7150/jca.46877

**Published:** 2021-01-01

**Authors:** Mei-Pan Yin, Peng-Fei Xie, Yue Zhao, Wei He, Yao-Zhen Ma, Chun-Xia Li, Zhen Li, Yan-Wei Zeng, Gang Wu

**Affiliations:** 1 Department of Interventional Radiology, The First Affiliated Hospital of Zhengzhou University, Zhengzhou 450052, China; 2 Oncology Department, The First Affiliated Hospital of Zhengzhou University, Zhengzhou 450052, China

**Keywords:** Esophageal cancer, Transarterial infusion chemotherapy, Complication, Interventional radiology

## Abstract

**Background**: Most esophageal cancer patients are diagnosed at an advanced stage when there are few effective treatments. Transarterial infusion chemotherapy is a local chemotherapy method wherein chemotherapeutic drugs are directly injected into tumor vessels.

**Methods**: Transarterial infusion chemotherapy was performed on advanced esophageal cancer patients once a month, and each patient underwent 1-3 treatments. The clinical results, complications, and effectiveness rates of each treatment episode were recorded and analyzed.

**Results**: Transarterial infusion chemotherapy was successfully performed in all patients, and no severe complications such as paraplegia or death were noted. Complete response, partial response, and stable disease were noted in 17.3% (13/75), 77.3% (58/75), and 5.3% (4/75) of cases after transarterial infusion chemotherapy, respectively. The total treatment efficacy (complete response + partial response) was 94.7%. All cases exhibited improvement in clinical stage, with a marked decrease in dysphagia. Subsequent treatments were administered to 13 patients, including radical radiation in 7 and chemotherapy in 6. During follow-up, death was caused by progressive carcinoma in 20, tumor-related pneumatic infection and respiratory failure in 11, and gastrointestinal hemorrhage in 17. The median survival time was 15 months and the 1-year survival rate was 58.1%.

**Conclusions**: Transarterial infusion chemotherapy may be safely and effectively used for treatment of advanced esophageal cancer.

## Introduction

Most patients with advanced esophageal cancer do not undergo vital radical surgery due to the lack of specific clinical manifestations and because of the limitations of imaging methods for early esophageal cancer 1. Moreover, the insensitivity of esophageal cancer cells to radiotherapy and chemotherapy limits their therapeutic effect 2. A majority of patients with advanced esophageal cancer die within a year after diagnosis (3), the 5-year survival rate ranges from 10% to 25%, and the overall prognosis is poor 4-6.

Arterial infusion chemotherapy is a local chemotherapy method wherein chemotherapeutic drugs are directly injected into tumor vessels by targeting blood vessels. Compared with traditional intravenous chemotherapy, arterial infusion chemotherapy can significantly increase the concentration of chemotherapeutic drugs in local tumor areas, reduce the damage to surrounding normal tissues, and reduce the toxicity and side effects of chemotherapeutic drugs. In 1977, Tanohata successfully used target artery infusion chemotherapy for esophageal cancer for the first time 7. The method improved the killing effect of cancer cells by generating high concentrations of drugs in the lesions. Due to the advantages of reduced side effects and improved tolerance, this method effectively improved the quality of life of patients. Kenzo Maruta et al. used bleomycin intra-arterial infusion chemotherapy to treat esophageal cancer, and found that bleomycin intra-arterial infusion chemotherapy after surgery increased the 5-year survival rate 8. In the present study, we aimed to evaluate the clinical efficacy and safety of conventional chemotherapy regimens in the treatment of esophageal cancer through arterial infusion.

## Materials and Methods

### Patients

We retrospectively analyzed the clinical data of patients with advanced esophageal cancer treated by arterial infusion chemotherapy. This study was approved by the Medical Record Management Department in our hospital. All patients signed informed consent forms.

Inclusion criteria: ① Age >18 yr, gender unlimited; ② Esophageal cancer confirmed by histopathology, advanced esophageal cancer confirmed by imaging data; ③ Patients with initial diagnosis or failure of radiochemotherapy or recurrence after surgery; ④ ECOG ≤2.

Exclusion criteria: ① Poor coagulation function, INR >1.5, or anticoagulant therapy or known bleeding disease; ② Unstable coronary heart disease or recent myocardial infarction (within 1 year); ③ Leukocytes <3000 cell/mm^3^ or platelet count <50000 /mm^3^; ④ Renal insufficiency (creatinine >2 mg/L); ⑤ Aspartate transaminase and/or alanine transaminase >5-times the normal upper limit; ⑥ Digital subtraction angiography (DSA) showed that the artery supplying the tumor contained the anterior spinal artery or the artery was too slender and the intubation was unsuccessful.

### Transarterial infusion chemotherapy

For treatment, the patient was conscious and was placed in the supine position on a DSA examination bed. The right femoral artery puncture point was locally anesthetized and a 5F sheath was inserted. The feeding artery of the tumor was identified via angiography using a 5F Cobra catheter or vertebral artery catheter through the sheath. Depending on the tumor location, we found that the corresponding target vessels had a clear distribution: (1) the bilateral inferior thyroid artery was the major supply vessel for carotid esophageal cancer; (2) the bilateral bronchial artery and/or esophageal artery was the major supply vessel for thoracic esophageal cancer, including the bilateral inferior thyroid artery as the major supply vessel for upper thoracic esophageal cancer and the esophageal artery and left gastric artery as the major supply vessel for lower thoracic esophageal cancer; and (3) the bilateral inferior thyroid artery, bilateral bronchial artery, left gastric artery, and/or gastroduodenal artery was the major supply vessel for recurrent esophageal cancer after surgery.

Based on the patient's body surface area and physical condition, epirubicin (30-50 mg), oxaliplatin (100 mg), and raltitrexed (4 mg) were administered, and a 150-200 ml diluted solution was prepared for each chemotherapeutic drug. According to the tumor blood supply, the perfusion dose of the chemotherapeutic drugs was appropriately distributed, and the perfusion time for each drug was set at 15-20 minutes.

Four weeks after transarterial infusion chemotherapy, a chest enhanced CT examination was performed to evaluate the therapeutic effect. If CR was achieved, the tumor was subsequently treated with radiotherapy or surgery. If PR or SD was achieved, arterial infusion chemotherapy was performed once more. If progressive disease (PD) was noted, the patient was switched to other palliative treatment.

### Evaluation of clinical efficacy and adverse reactions

Routine blood tests, liver and kidney function tests, and blood coagulation function tests were performed before and after the surgery. Esophagography and chest CT were performed before treatment, 1 month after treatment, and 2 months after treatment. According to new guidelines for evaluating the efficacy of treatment of solid tumors 9,10, the clinical efficacy of esophageal cancer treatment was determined as complete remission (CR), partial remission (PR), unchanged (SD), or progressive disease (PD). The total efficacy was assessed based on the proportion of CR+PR cases.

Adverse drug reactions, changes in tumor size, and laboratory results were recorded. The survival time of patients was determined, and the toxicity and side effects were evaluated based on National Cancer Institute Common Terminology Criteria for Adverse Events (NCI-CTCAE, version 4.0) and the toxicity grading (0-IV) of anticancer drugs.

### Statistical analysis

All data were analyzed by GraphPad Prism 5 software. An independent double sample t test was used to compare quantitative data, and the results are presented as mean ± standard deviation. Continuous variables were compared using a t-test, and classified data were compared using the chi-square test. A P value <0.05 is considered to indicate statistical significance.

## Results

### Patient characteristics

In this study, we enrolled 75 patients, including 54 men and 21 women with a mean age of 65.9±8.3 years (range, 48-89 years). With regard to histological type, 73 patients had squamous cell carcinoma (97.3%) and 2 patients had adenocarcinoma (2.7%). With regard to location, lesion was noted in the upper esophageal cancer, upper and middle, middle, middle and lower, lower, and anastomotic site in 15, 9, 16, 6, 5, and 24 cases, respectively (Table 1). Prior to surgery, hypertension, diabetes, cardiac disease, and other conditions were noted in 19, 5, 7, and 13 cases, respectively.

Complications such as preoperative esophageal fistulas were noted in 15 cases, including 13 cases of esophagotracheal fistula. Esophagomediastinal fistula and esophagopleural fistula were noted in 1 case each. Among the cases of esophageal fistula, 7 were occluded with fully covered stents. The other cases underwent jejunostomy tube placement and conservative therapy. Moreover, 13 cases received subsequent treatment, including 7 cases who received radiotherapy and 6 cases who received systematic chemotherapy.

### Operation and adverse effects

All the patients successfully underwent TAI chemotherapy on a mean of 2.22±0.07 arteries (range, 1-3 arteries). In particular, the procedure was performed 16 times on the bilateral inferior thyroid artery, 11 times on the unilateral inferior thyroid artery, 42 times on the bilateral bronchial artery, 35 times on the unilateral bronchial artery, 20 times on the esophageal artery, 14 times on the intercostal artery, 18 times on the right gastroepiploic artery, and 2 times on the left gastric artery and right gastric artery. Superselective catheterization with a microcatheter was performed in 36 cases to protect the blood vessels, avoid the spinal artery, and prevent drug reflux. Mild laryngeal edema was noted in 1 patient during the operation. All patients exhibited symptom relief after treatment, and no serious complications such as paraplegia or operation-related death were noted.

Postoperative adverse reactions with grades I-IV were observed, which were alleviated over a short time (Table 2). Nausea and vomiting, hemoglobin level decrease, thrombocytopenia, fever, liver failure, leukopenia, and other common adverse reactions were noted in 45 cases (60.0%), 31 cases (41.3%), 16 cases (21.3%), 12 cases (16.0%), 5 cases (6.7%), and 9 cases (12.0%), respectively.

### Evaluation of the clinical efficacy

**Fig 1:**
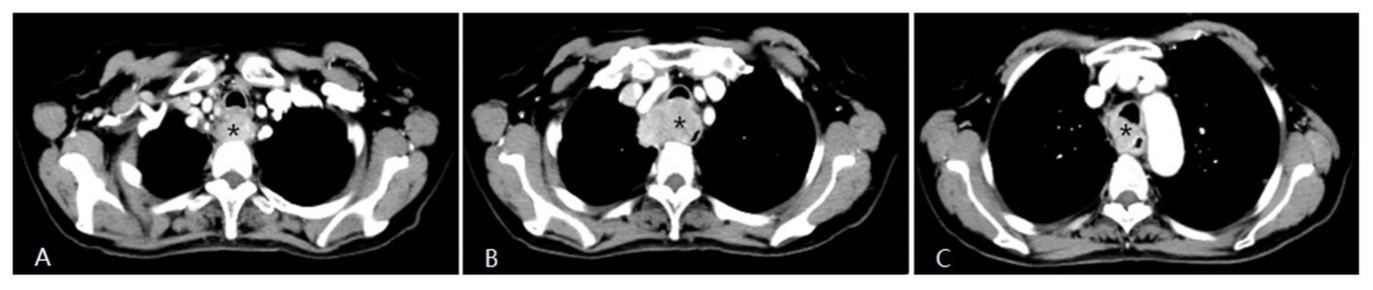
A patient with progressive dysphagia for 2 months was diagnosed with esophageal squamous cell cancer via endoscopic biopsy. Due to her poor condition, the oncologists did not recommend systemic chemotherapy, but instead suggested arterial infusion chemotherapy. Chest CT shows an apparently enhanced mass (*) in the middle-upper esophagus with moderate stenosis of the tracheal cavity due to tumor invasion.

**Fig 2:**
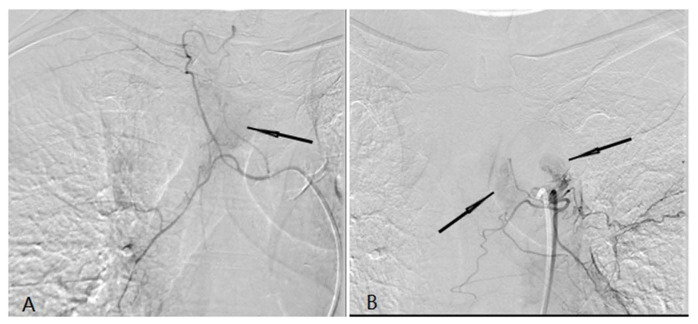
A tumor feeding artery derived from the right tracheal artery and there was abnormal tumor staining (black arrow) (A). (b) A tumor feeding artery derived from the left tracheal artery and there was abnormal tumor staining (black arrow) (B).

**Fig 3:**
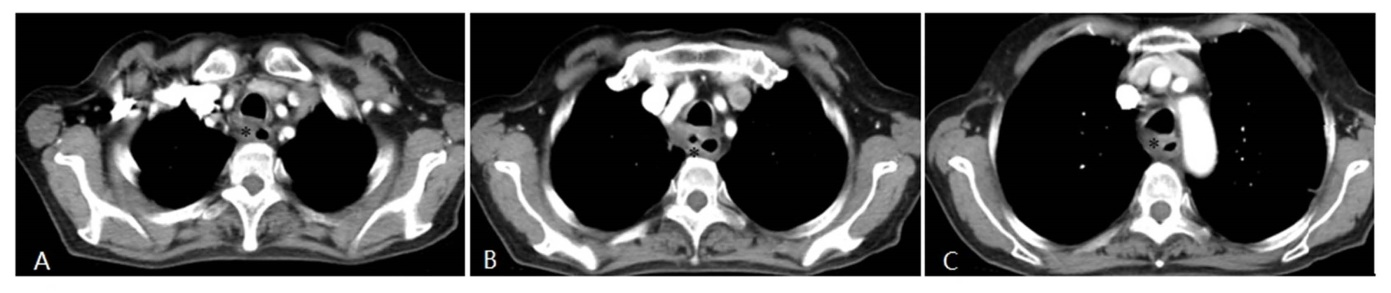
Four weeks after interventional therapy, a chest CT scan shows that the lesion has significantly reduced, tracheal lumen is normal, and the arterial phase enhancement is not apparent (*) (A, B, C).

According to Stooler's classification criteria for dysphagia, we originally defined the patients with esophageal fistula with a nasojejunal feeding tube before operation as grade 4 in this study. A total of 43 patients had grade 3 dysphagia, 32 patients had grade 4 dysphagia, and 15 patients had a preoperative esophageal fistula. Seven patients with an esophageal fistula were treated with a covered esophageal stent, whereas the remaining underwent nasojejunal feeding tube placement. One patient with a preoperative esophagotracheal fistula underwent nasojejunal feeding tube placement and 2 regimens of transcatheter arterial infusion (TAI) chemotherapy, and the fistula completely healed. In 8 patients, the nasojejunal feeding tube was extracted 1 week after arterial infusion chemotherapy, and the patients were allowed to orally ingest food thereafter. Moreover, 5 patients (2 with CR and 3 with PR) had an esophageal fistula after the operation, which was considered to be due to the rapid reduction of the tumor after operation, resulting in insufficient repair of the surrounding normal tissue. A total of 33 patients had dyspnea before the operation. Among these 33 patients, 20 with severe airway stenosis underwent airway stent insertion (11 tubular stents and 9 Y-shaped stents). After TAI chemotherapy, the tumors in the other cases shrank, and the symptoms of tracheal compression or invasion were alleviated without any need for further treatment (Figure 1-3).

Based on the clinical stages of the American Joint Cancer Commission (AJCC) 11, 1, 29, 6, and 39 cases were categorized as T2, T3, T4a, and T4b, respectively. After treatment, 18, 32, 15, 0, and 10 cases were categorized as T1, T2, T3, T4a, and T4b, respectively. Thus, there was a significant decrease in the clinical stage after treatment (Table 3).

As shown in Table 4, 13, 58, and 4 cases exhibited CR, PR, and SD after the first TAI. The total objective response rate (CR+PR) was 96.0%. Thereafter, 21 patients received a second TAI and 3 patients received a third TAI. After the second operation, 5, 14, and 2 cases exhibited CR, PR, and SD, respectively. The total objective response rate (CR+PR) was 88.9%. Finally, after 1-3 courses of treatment, 13 cases of CR, 58 cases of PR, and 4 cases of SD were followed. The total objective response rate (CR+PR) was 94.7%.

### Follow-up and survival time

Follow-up was performed via phone interviews or in-person interviews at the clinic. During the follow-up period, 20 patients died of functional failure at the terminal stage of the cancer, 11 patients died of pneumatic infection and respiratory failure associated with the cancer, and 17 patients died of gastrointestinal bleeding. Of the 27 patients that survived, 4 had esophagotracheal fistula and underwent nasojejunal feeding tube placement. The median survival time was 15 months, and the 1-year survival rate was 58.1%.

## Discussion

Due to the advances in surgical methods and techniques for esophageal cancer, surgical trauma and postoperative recovery have improved significantly. However, due to the persistence of residual cancer and the metastasis of distant small lesions, the prognosis of esophagectomy remains poor; in fact, the 5-year survival rate is only 15-20%. In recent years, the comprehensive treatment of esophageal cancer has been attempted, including preoperative neoadjuvant chemotherapy, neoadjuvant radiotherapy, neoadjuvant radiotherapy, and chemotherapy 12.

Some studies 12 have shown that preoperative neoadjuvant radiotherapy can prolong the disease-free survival time of patients after surgery and can improve the local control rate. Hence, patients can benefit from the longer survival time. However, other studies have found that the treatment does not prolong the overall survival time. For patients undergoing surgical treatment after radiation, such treatment may increase the incidence of postoperative complications and mortality.

Furthermore, many studies have found that preoperative neoadjuvant chemotherapy can reduce the clinical stage of esophageal cancer, control the small distant metastasis of tumor cells, and improve the disease-free survival time after surgery. Cisplatin combined with fluorouracil is the first-line regimen for the systematic chemotherapy of esophageal cancer. With the progression and advent of new chemotherapeutic agents and clinical needs, new combined regimens can be considered synchronously. Studies have shown that the EOF regimen (epirubicin+oxaliplatin+5-Fu) is effective for advanced esophageal cancer, with a 1-year survival rate of 40.4%. However, due to the limitations of the toxicity and side effects of chemotherapeutic drugs, patients' general condition, and resistance of cancer cells, the effective rate of chemotherapy for esophageal cancer is only 15-53% 13,14.

Yabei Liu et al. 15 reported 46 patients with esophageal cancer were treated with target artery perfusion of verapamil and chemotherapy; there were a total of 2 (4.3%) cases of CR, 39 (84.8%) cases of PR, 3 (6.5%) cases of SD, and 2 (4.3%) cases of PD. The overall objective response rate (CR + PR) was 89.13%. In the present study, 75 patients with advanced esophageal cancer were treated with TAI chemotherapy, there were a total of 13 (17.3%) cases of CR, 58 (77.3%) cases of PR, and 4 (5.3%) cases of SD. The overall objective response rate was 94.7%.

Accurate and comprehensive search for the feeding arteries of esophageal cancer is vital for achieving favorable outcomes following arterial infusion chemotherapy. The arteries supplying esophageal cancer are complex and may differ. Failure to identify or overlooking the supplying arteries may affect the clinical efficacy. In the present study, all patients successfully underwent infusion into the tumor feeding artery. Based on the angiographic DSA findings and preoperative imaging results, the blood supply covers the entire tumor to obtain satisfactory results.

In conclusion, TAI chemotherapy is safe and effective for advanced esophageal cancer. However, this study is a single-center retrospective analysis, and may hence have a choice bias. In the future, we will perform a multi-center, large-sample prospective study to obtain more objective and sufficient evidence.

## Figures and Tables

**Table 1:** Patient characteristics

Categories	Number of cases
Patients, No.	75
Gender, male	54(72%)
Mean age, years	65.9±8.3
Histological types	
Squamous cell carcinoma	73 (97.3%)
Adenocarcinoma	2(2.7%)
Location of cancer	
Upper segment	15 (20%)
Upper and middle segment	9(12%)
Middle segment	16(21%)
Lower and middle segment	6 (8%)
Lower segment	5 (7%)
Anastomotic segment	24(32%)
Comorbidities, No. (%)	
Hypertension	19 (25%)
Diabetes mellitus	5 (7%)
Heart disease	7(9%)
Others	13 (17%)

**Table 2:** Adverse effects in patients with interventional treatment.

Stage	I	II	III	IV
WBC count decrease	2	1	5	1
Hemoglobin level decrease	18	7	6	0
Platelet count decrease	11	3	2	0
ALT/AST level increase	4	1	0	0
Nausea and vomiting	34	11	0	0
Fever	4	4	4	0

WBC, white blood cell; ALT, alanine transaminase; AST, aspartate transaminase.

**Table 3:** Clinical stage before and after interventional treatment.

Stage	Before treatment	After the first treatment	After the second treatment	After the third treatment
n	75	75	21	3
T1	0	18***	11***	1
T2	1	32***	6***	1
T3	29	15*	3*	0
T4a	6	0	0	0
T4b	39	10***	1***	1

*p<0.05, **p<0.01, ***p<0.001.

**Table 4:** Clinical outcome and follow up.

	After first TAI	After second TAI	After third TAI	During follow up
n	75	21	3	75
CR	8 (10.7%)	5(23.8%)	1(33.3%)	13(17.3%)
PR	64(85.3%)	14(66.7%)	1(33.3%)	58(77.3%)
SD	3(4.0%)	2(9.5%)	1(33.3%)	4(5.3%)
PD	0	0	0	0
CR+PR	72(96.0%)	19 (88.9%)	2(66.7%)	71(94.7%)

CR: Complete Response; PR: Partial Response; SD: Stable Disease; PD: Progressive Disease

## Abbreviations

TAI: Transarterial infusion chemotherapy
CT: Computed tomography
DSA: Digital subtraction angiography
CR: Complete response
PR: Partial response
SD: Stable disease
PD: Progressive disease
AJCC: American Joint Cancer Commission
NCI- CTCAE: National Cancer Institute Common Terminology Criteria for Adverse Events
